# Distribution characteristics of chronic fatigue syndrome among secondary school students and its association with physical health: a case study in Shaanxi Province

**DOI:** 10.3389/fped.2026.1821254

**Published:** 2026-07-06

**Authors:** Yuan Yuan, Li Wen, Hongli Fu, Yinzhong Ren, Yiming Ren, Xiaodong Liu, Fang Chen, Zhimei Shen, Feng Wang, Aiping Chi, Xiaoxiong He

**Affiliations:** 1 School of Physical Education, Shaanxi Normal University, Xi'an, Shaanxi, China; 2 Chengdu Aerospace High School, Chengdu, Sichuan, China; 3 Weinan Jicai Middle School, Weinan, Shaanxi, China; 4 Yankou Town Nine-Year Compulsory Education School, Hanzhong, Shaanxi, China; 5 Shaanxi University of Technology, Hanzhong, Shaanxi, China

**Keywords:** adolescents, chronic fatigue syndrome, mental health, physical fitness test, secondary school students

## Abstract

**Objective:**

The aim of this study is to examine the detection rate of chronic fatigue syndrome (CFS) in secondary school students in Shaanxi Province and its association with their physical fitness test outcomes.

**Methods:**

A survey involving the distribution of CFS questionnaires and collection of physical fitness test data was carried out among students from 25 secondary schools in Shaanxi Province. A total of 8,840 valid questionnaires and datasets were analyzed. Detection rates for CFS and chronic fatigue (CF) were determined based on screening criteria and severity levels. Gender and year group distribution variances for CFS and CF were assessed using chi-square tests, while correlation analyses were performed to evaluate the relationship between CFS and physical fitness test scores.

**Results:**

Results showed an overall CFS detection rate of 2.059% among students, with rates of 1.903% for males and 2.234% for females. There was no significant difference in CFS detection rates between genders (*χ*^2^ = 1.210, *p* = 0.546). The CFS detection rate increased from 1.012% in grade 7 to 3.728% in the Cram school, with significant differences across grade groups (*χ*^2^ = 24.082, *p* = 0.020). Correlation analysis revealed a weak to moderate negative correlation between CFS severity and performance grades in the 1,000-meter run for boys (*τ* = −0.261, *p* < 0.001) and the 800-meter run for girls (*τ* = −0.385, *p* < 0.001). Among female students in cram schools, those in the CFS group had significantly lower vital capacity than the control group (*p* < 0.05), whereas no significant difference was found between the CF group and the control group. Both the CF and CFS groups exhibited significantly lower 800 m or 1,000 m running test results than control group students to varying degrees.

**Conclusion:**

CFS-like symptoms among secondary school students in Shaanxi Province warrant attention. The screening detection rate tended to increase with grade level and was highest among students in cram schools. Higher CFS levels were associated with poorer physical fitness achievement, particularly endurance running performance, suggesting that students with CFS-like symptoms may require additional attention in school health monitoring.

## 1 Introduction

Chronic Fatigue Syndrome (CFS) constitutes a severe sub-health condition characterised by persistent, long-term fatigue, accompanied by symptoms such as low-grade fever, headaches, impaired concentration, memory decline, sleep disturbances, and depression (1), whilst physical examinations and routine tests generally reveal no significant abnormalities (2). CFS has been associated with individual, familial, and societal factors (3, 4). Secondary school pupils are at a critical stage of psychological development and cognitive growth; the persistently high-pressure learning environment may be linked to the mental health and physical well-being of some pupils (5). A survey conducted by Chinese researchers indicates that the positive detection rate for chronic fatigue syndrome among secondary school students in Suzhou is approximately 1.0% (6). Another study suggests that the positive detection rate for chronic fatigue syndrome among secondary school students in Shanghai is approximately 21.1% (7), highlighting the substantial scale of this condition within China's secondary school cohort. In recent years, the implementation of the national “Double Reduction” policy for primary and secondary school pupils has alleviated academic pressures to some extent. Concurrently, the Ministry of Education has mandated enhanced physical exercise and health monitoring (8), resulting in noticeable improvements in pupils' physical wellbeing after several years of effort. However, some studies suggest that engaging in vigorous exercise whilst in poor physical condition or suffering from chronic fatigue not only reduces athletic ability and performance but also increases the risk of sports injuries (9, 10). Notably, animal experiments have confirmed that CFS is a contributing factor to sudden death following high-intensity exercise (11), indicating that the coexistence of CFS and vigorous physical exertion can inflict more severe damage on the body.

Although there have been international studies on CFS in the general population, data on the distribution of CFS symptoms and their association with physical fitness among Chinese adolescents—particularly within the current educational policy context—remain scarce. The Physical Fitness Test is a series of assessments developed by the Chinese Ministry of Education and the General Administration of Sport to evaluate the physical fitness of the general population. It comprises three components: body composition, physical function, and physical fitness. The results of the Physical Fitness Test for secondary school students serve as a key basis for evaluating their physical health (12). The primary symptoms of CFS include prolonged physical or mental fatigue and a general feeling of being unwell. Research has confirmed that individuals with CFS exhibit disturbances in their cardiopulmonary function and energy metabolism during high-intensity exercise (13), whereas certain middle-distance events (such as the 1,000-meter run) rely primarily on the body's oxygen transport system. The 1,000-meter run for boys and the 800-meter run for girls required by China's National Student Physical Fitness Standards are both high-intensity exercises involving a combination of aerobic and anaerobic metabolism (14). However, there is currently no evidence to suggest a correlation between CFS and performance on these “physical fitness test” events. This study aims to conduct an exploratory investigation into the distribution of CFS among secondary school students in Shaanxi Province, China, and the extent of its association with their physical health, using CFS questionnaire surveys and physical fitness tests. The findings will provide a theoretical basis for further improving the management of chronic fatigue syndrome and enhancing the physical health of secondary school students.

## 2 Methods

### 2.1 Survey population

The study utilized a stratified cluster sampling method to choose schools and classes for the survey. Schools were classified into four types based on operational models of Chinese secondary schools: model secondary schools, general secondary schools, vocational secondary schools, and private senior high school cram schools. Generally, model secondary schools are typically provincial or municipal key institutions, while general secondary schools are commonly public or private comprehensive establishments. Vocational secondary schools concentrate primarily on vocational education, and private high school cram schools are designed for students who have finished high school. Nonetheless, they also accommodate students who did not gain admission to their preferred university and intend to retake the college entrance exam, commonly referred to as “Cram schools”.

Based on the above stratification, 25 schools in Shaanxi Province were randomly selected as survey units, including 8 model secondary schools, 12 general secondary schools, 3 vocational secondary schools, and 2 cram schools. Next, at the class level within each selected school, a random cluster sampling method was employed: in the junior high school division, one class each was randomly selected from the 7th, 8th, and 9th grades (for a total of 3 classes); from the high school division, two classes were randomly selected from each of the 10th, 11th, and 12th grades (for a total of 6 classes); and from the cram schools, which do not have grade divisions, two classes were randomly selected. Through these two-stage sampling procedures, the sample for this study is well-represented in terms of both geographical distribution (within Shaanxi Province) and grade distribution (from 7th grade through 12th grade and cram classes).

In addition, prior to the survey, all participants were informed of the content, purpose, and important considerations of the study and signed informed consent forms. This study was approved by the Academic Ethics Committee of Shaanxi Normal University (No. 202516063).

### 2.2 Questionnaire survey method

Survey Questionnaire Selection: This study employed the US Centers for Disease Control and Prevention (CDC-1994) CFS questionnaire (6); Screening criteria for CFS: In addition to simultaneously meeting the criteria of “① unexplained fatigue persisting for more than three months” and “② fatigue that is not alleviated by rest”, individuals must also meet four or more of the following eight quantitative symptom indicators: “marked decline in concentration, sore throat, tenderness in the armpits, muscle pain, joint pain, headache, non-restorative sleep, and fatigue persisting for 24 h after activity” to be classified as having Chronic Fatigue Syndrome (CFS); Individuals meeting only 1–3 of these quantitative criteria are classified as having Chronic fatigue (CF); all others are classified as the control group (6, 15). In this study, the positive rate of the questionnaire screening is expressed as the “detection rate”.

Questionnaire distribution and collection: Questionnaires were distributed and collected between September 2023 and February 2024. A total of 10,350 questionnaires were distributed, with 9,631 returned, yielding a response rate of 93.05%. Among these, 8,840 were deemed valid, representing an effective rate of 85.41%. Questionnaire Validity Assessment: An exploratory factor analysis (EFA) was conducted on the entire sample (*N* = 8,840). The Kaiser-Meyer-Olkin (KMO) coefficient was 0.830, and Bartlett's sphericity test was significant (*p* < 0.001), confirming the adequacy of the sample. Regression analysis demonstrated significant equation coefficient regression effects with good model fit, and factor loadings of 0.65 confirmed the questionnaire's robust construct validity. Questionnaire Reliability Assessment: Cronbach's *α* coefficient (0.937) was employed to evaluate internal consistency reliability, indicating robust reliability of the questionnaire scale.

### 2.3 Testing methodology

For all selected survey participants, physical fitness test data for the current academic year was retrieved through their respective school physical education departments. For students from cram schools participating in this study, physical fitness assessments were conducted by the research team's faculty and students. All assessors underwent specialised training. Testing instruments were nationally standardised equipment for secondary school physical fitness assessments. Test items, scoring criteria, and performance grading were established in accordance with the National Student Physical Fitness Standards (2014 Revision) (16) issued by the Ministry of Education and the General Administration of Sport of China.

### 2.4 Statistical analysis

Data analysis and statistical processing were performed using SPSS 27 software. Descriptive analysis examined the distribution of CFS among students at varying severity levels. The Chi-square test (*χ*^2^) analyzed CFS distribution differences by gender and grade. Kendall's correlation test explored the relationship between CFS distribution at different severity levels and students' performance test results. Normality of performance test data in each group was assessed using the Shapiro–Wilk test. If data met normality assumptions, one-way ANOVA was used for comparison; otherwise, the Kruskal–Wallis test was employed. Results were presented as median M (P25, P75) using Kruskal–Wallis H test. Pairwise comparisons were conducted with the Dunn test when the overall test was significant (*P* < 0.05), adjusting the significance level with the Bonferroni correction. Statistical significance was determined only when the Bonferroni-corrected *P* value was below 0.05.

### 2.5 Quality control

Standardised training shall be provided for postgraduate and undergraduate students participating in questionnaire surveys and physical fitness assessments. During questionnaire administration, on-site guidance for form completion shall be conducted in collaboration with form tutors of sampled classes. Collected questionnaires shall undergo rigorous review, with incomplete or ambiguously completed forms excluded to ensure the scientific validity and accuracy of research data. For physical fitness assessments, calibrate testing equipment in advance and strictly adhere to national student physical fitness testing protocols and requirements. Both questionnaire and test data shall undergo dual data entry using EpiData 3.1 software. Discrepancies identified through consistency checks shall be verified against original records to ensure data entry accuracy.

## 3 Results

### 3.1 Distribution characteristics of CFS and CF Among secondary school students

The comparison of CFS and CF detection rates among secondary school students by year group and gender is presented in Table 1. Results indicate that the overall detection rates for CFS and CF among secondary school students were 2.059% and 8.314% respectively. Specifically, the CFS detection rates for male and female students were 1.903% and 2.234% respectively, while the CF detection rates were 8.296% and 8.335% respectively. No significant differences were observed in the gender distribution of CFS or CF (*χ*^2^ = 1.210, *p* = 0.546). Furthermore, comparisons of CFS detection rates across different educational stages revealed an increasing trend from grade 7 to cram schools, with significant differences observed between grade levels (*χ*^2^ = 24.082, *p* = 0.020). Notably, the CFS detection rate among cram school students reached 3.728%. The distribution tendencies of CFS and CF across different grades revealed that while CF distribution showed no significant tendency, CFS distribution exhibited a marked tendency towards cram schools rather than Grade 7. These findings suggest that attention should be paid to student CFS distribution starting from Grade 8, with particular emphasis required for students in cram schools.

**Table 1:** Distribution characteristics of CFS in secondary school students (*n* = 8,840).

Demographic indicators		CFS		CF		*χ* ^2^	*p*
		Detection rate (%) (n)	Adjusted residuals*^Δ^*	Detection rate (%) (n)	Adjusted residuals*^Δ^*		
Grade	Grade7	1.012 (8)	‒**2**.**2**	7.098 (56)	‒1.3	24.082	0.020*
	Grade8	1.689 (15)	‒0.8	6.532 (58)	‒2.0		
	Grade9	1.843 (15)	‒0.5	7.371 (60)	‒1.0		
	Grade10	2.179 (39)	0.4	9.050 (162)	1.3		
	Grade11	2.030 (40)	‒0.1	8.376 (165)	0.1		
	Grade12	2.130 (42)	0.3	8.874 (175)	1.0		
	Cram school	3.728 (23)	**3**.**0**	9.562 (59)	1.2		
Gender	Male	1.903 (89)	—	8.296 (388)	—	1.210	0.546
	Female	2.234 (93)	—	8.335 (347)	—		
Overall		2.059 (182)	—	8.314 (735)	—	—	—

^Δ^ The criterion for judging the distribution tendency of varying degrees of CFS within each year group is: adjusted standardised residual absolute value >2.Bold values indicate adjusted standardized residuals with absolute values >2, denoting a significant distribution tendency.

—, Not applicable to this statistical indicator.^*^p < 0.05 (There are differences in the detection rate of CFS among different grades).

**Figure 1:**
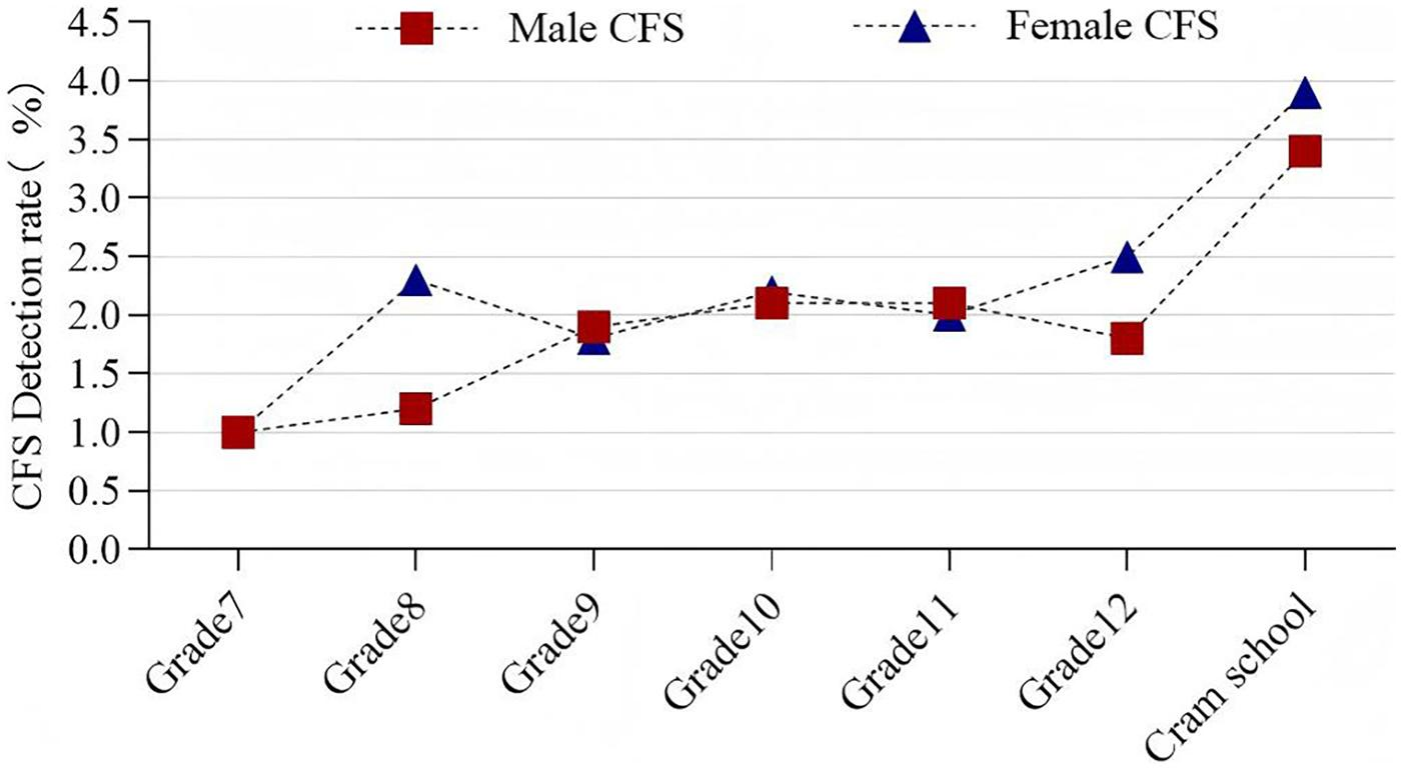
Trends in CFS detection rates Among male and female students across different year groups.

The trends in CFS detection rates for male and female students are shown in Figure 1. Results indicate that CFS detection rates increase with grade level for both genders, particularly among students in cram schools where rates rise significantly to 3.9% for females and 3.4% for males.

### 3.2 Correlation between CFS and physical fitness test grading levels in secondary school students

To identify which physical fitness test items were associated with CFS levels, this study conducted Kendall's tau-b correlation analysis between students' CFS levels (control, CF, CFS) and their physical fitness test grading levels (Fail, Pass, Good, Excellent). Statistical results are presented in Table 2.

**Table 2:** Correlation test results between students’ CFS levels and physical fitness test achievement levels.

Test items	Male (*n* = 4,677)				Female (*n* = 4,163)			
	*τ*	95% CI	*Z*	*p*	*τ*	95% CI	*Z*	*p*
BMI	−0.002	−0.030, 0.026	−0.17	0.869	−0.082	−0.111, −0.052	−5.48	<0.001
Vital capacity	−0.030	−0.056, −0.003	−2.19	0.029	−0.032	−0.060, −0.004	−2.24	0.025
50 m	−0.012	−0.040, 0.01	−0.90	0.371	**−**0.098	−0.126, −0.070	−6.81	<0.001
Sit and Reach	−0.007	−0.036, 0.022	—0.47	0.637	−0.028	−0.056, 0.004	−1.93	0.053
Standing Long Jump	−0.040	−0.066, −0.014	−2.95	0.003	−0.121	−0.148, −0.094	−8.44	<0.001
Pull-ups	−0.018	−0.046, 0.009	−1.28	0.202	—	−	—	—
1 min Sit-ups	—	−	—	—	−0.094	−0.124, −0.065	−6.24	<0.001
1,000 m	**−0**.**261**	−0.287, −0.235	−**19**.**51**	<**0**.**001**	—	−	—	—
800 m	—	−	—	—	**−0**.**385**	−0.413, −0.357	−**26**.**82**	<**0**.**001**

—, This test item is not specified.Bold values represent moderate-to-strong correlations (| *τ* | ≥ 0.2).

By employing Kendall's tau-b correlation analysis, the research explored the relationship between CFS levels and physical test scores in male and female students. Elevated CFS levels were generally associated with lower physical test scores, with most correlations demonstrating a relatively weak strength. Among male students, chronic fatigue syndrome (CFS) levels exhibited weak negative correlations with vital capacity achievement level (*τ* = −0.030, *Z* = −2.19, *p* = 0.029) and standing long jump achievement level (*τ* = −0.040, *Z* = −2.95, *p* = 0.003). These correlations, however, were of minimal magnitude. No statistically significant correlations were found for BMI, 50-m sprint, sit-and-reach, or pull-ups. Notably, the most substantial correlation among male students was observed for the 1,000-m run, where CFS levels displayed a weak-to-moderate negative correlation with achievement level (*τ* = −0.261, *Z* = −19.51, *p* < 0.001).

In female students, levels of CFS were inversely correlated with BMI achievement level (*τ* = −0.082, *Z* = −5.48, *p* < 0.001), vital capacity achievement level (*τ* = −0.032, *Z* = −2.24, *p* = 0.025), 50-m sprint achievement level (*τ* = −0.098, *Z* = −6.81, *p* < 0.001), standing long jump achievement level (*τ* = −0.121, *Z* = −8.44, *p* < 0.001), and 1-min sit-up achievement level (*τ* = −0.094, *Z* = −6.24, *p* < 0.001). There was no statistically significant association found for sit-and-reach (*τ* = −0.028, *Z* = −1.93, *p* = 0.053). The most substantial correlation among female students was observed with the 800-m run, displaying a moderate negative correlation with CFS levels (*τ* = −0.385, *Z* = −26.82, *p* < 0.001).

The collective results suggest that higher CFS levels were linked to decreased levels of physical fitness achievement, particularly in endurance running events. However, aside from the 1,000-m run for boys and the 800-m run for girls, most statistically significant correlations were of small magnitude and should be interpreted with care.

### 3.3 Comparison of student performance in aerobic endurance-related physical fitness test items

Based on the aforementioned correlation results, this study further grouped and compared scores from physical side items showing higher correlations with CFS. Selected item scores included the 1,000 m run for males, the 800 m run for females, and vital capacity indicators related to aerobic endurance. The comparison results are presented in Tables 3, 4, 5 and 6.

**Table 3:** Comparison of students’ vital capacity (mL) test results

Gender	Grade	Control Group	CF Group	CFS Group	*H*	*p*	*η* ^2^
Male	Grade7	2,851.00 (2,186.10, 3,449.00)	2,087.00 (1,935.00, 2,905.00)	2,297.50 (2,032.50, 3,210.50)	4.234	0.12	0.006
	Grade8	2,801.00 (2,173.50, 3,703.00)	2,818.00 (2,299.50, 3,125.00)	2,621.00 (2,146.00, 2,845.00)	0.908	0.635	0
	Grade9	2,941.00 (2,254.50, 3,647.50)	2,589.00 (2,208.50, 3,588.50)	2,984.50 (2,318.50, 3,536.50)	0.666	0.717	0
	Grade10	2,996.50 (2,277.00, 3,666.00)	2,965.00 (2,209.50, 3,691.00)	2,410.00 (2,079.00, 3,254.00)	2.653	0.265	0.001
	Grade11	3,006.50 (2,244.50, 3,708.00)	3,000.00 (2,205.00, 3,456.00)	3,038.00 (2,432.50, 3,519.00)	0.515	0.773	0
	Grade12	2,964.00 (2,258.50, 3,673.00)	2,842.00 (1,951.00, 3,810.00)	2,654.50 (2,039.50, 3,213.50)	3.595	0.166	0.001
	Cram School	2,655.50 (2,018.00, 3,572.00)	2,534.00 (1,911.00, 4,001.00)	2,541.00 (1,787.00, 3,450.00)	0.483	0.785	0
Female	Grade7	1,940.50 (1,648.00, 2,255.50)	2,055.00 (1,644.00, 2,897.00)	1,795.00 (1,568.00, 1,892.00)	2.46	0.292	0.001
	Grade8	2,461.00 (2,024.50, 3,069.00)	2,090.00 (1,812.00, 2,584.00)	2,920.00 (1,978.00, 3,641.00)	4.928	0.085	0.008
	Grade9	2,378.00 (2,044.00, 2,904.50)	2,155.00 (1,974.00, 2,526.00)	2,367.00 (1,932.00, 3,169.00)	5.187	0.075	0.008
	Grade10	2,217.00 (1,847.00, 2,618.00)	2,324.00 (2,106.00, 2,657.00)	2,480.00 (2,105.00, 3,011.00)	6.183	0.045*	0.005
	Grade11	2,307.00 (1,964.00, 2,741.00)	2,251.50 (2,048.50, 2,517.50)	2,106.50 (1,722.00, 2,774.50)	2.642	0.267	0.001
	Grade12	2,225.50 (1,818.00, 2,714.50)	2,125.00 (1,934.00, 2,503.00)	2,165.00 (1,904.00, 2,881.00)	1.445	0.485	0
	Cram School	2,456.00 (2,156.00, 2,879.00)	2,307.00 (1,968.00, 2,475.00)	2,136.00 (1,889.00, 249.00)	12.086	0.002**	0.027

Data comparisons were performed using the Kruskal–Wallis *H* test, with results expressed as the median M (P25, P75), When compared with the control group.

* *p* < 0.05.

** *p* < 0.01.

**Table 4:** Pairwise comparisons of vital capacity (mL) among female students by grade (Dunn's test with Bonferroni correction)

Gender	Grade	Control VS CF			Control VS CFS			CF VS CFS		
		*Z*	*P*	95%CI	*Z*	*P*	95%CI	*Z*	*P*	95%CI
Female	Grade10	10.676	<0.001***	(−11, 265)	6.504	0.001***	(−26, 572)	0.543	1	(−158, 467)
	Cram School	6.566	<0.001***	(−394, −78)	5.436	<0.001***	(−617, −25)	0.969	0.997	(−371, 229)

Pairwise comparisons were conducted using Dunn's test with Bonferroni adjustment, Bonferroni-adjusted *p* values are reported. *p*_adj < 0.05 was considered statistically significant. When compared with the control group.

*** p(adj) < 0.001.

**Table 5:** Comparison of students’ 1,000/800 m(s) test results

Gender	Grade	Control Group	CF Group	CFS Group	*H*	*p*	*η* ^2^
Male	Grade7	259.00 (241.00, 275.00)	299.00 (286.00, 368.00)	321.00 (296.00, 323.00)	54.62	<0.001***	0.133
	Grade8	250.00 (241.00, 268.00)	289.00 (259.00, 373.00)	309.50 (306.00, 312.00)	45.327	<0.001***	0.088
	Grade9	245.00 (233.00, 274.00)	263.00 (221.50, 350.00)	298.00 (296.50, 300.00)	24.17	<0.001***	0.054
	Grade10	242.00 (215.00, 274.00)	274.00 (252.00, 332.50)	294.00 (288.50, 304.00)	83.391	<0.001***	0.088
	Grade11	238.00 (219.00, 265.00)	262.00 (240.00, 320.00)	286.00 (242.00, 303.00)	60.558	<0.001***	0.053
	Grade12	241.00 (217.00, 266.00)	258.00 (215.00, 316.00)	277.00 (248.00, 291.50)	22.35	<0.001***^,#^	0.019
	Cram School	246.50 (222.00, 268.00)	270.00 (225.00, 308.00)	283.50 (253.50, 289.50)	5.421	0.066	0.015
Female	Grade7	225.00 (221.00, 228.00)	275.00 (263.00, 288.00)	300.00 (298.50, 320.50)	73.846	<0.001***	0.186
	Grade8	221.00 (217.00, 225.00)	277.00 (261.50, 301.00)	315.00 (305.00, 328.00)	89.261	<0.001***	0.224
	Grade9	227.00 (215.00, 243.00)	253.00 (231.00, 275.00)	267.00 (265.00, 271.00)	24.783	<0.001***	0.057
	Grade10	223.00 (213.00, 240.00)	282.50 (261.00, 298.00)	294.00 (283.00, 304.00)	175.344	<0.001***	0.203
	Grade11	225.00 (217.00, 245.00)	241.50 (220.50, 254.50)	283.00 (250.50, 303.50)	128.89	<0.001***^,###^	0.148
	Grade12	220.00 (207.50, 241.00)	242.50 (226.00, 265.00)	280.00 (237.00, 288.00)	94.771	<0.001***^,#^	0.106
	Cram School	229.00 (215.00, 251.00)	256.00 (249.00, 266.00)	280.00 (230.00, 291.00)	45.954	<0.001***	0.116

Data comparisons were performed using the Kruskal–Wallis H test, with results expressed as the median M (P25, P75), When compared with the control group.

*** *p* < 0.001; compared with the CF group.

# p(adj) < 0.05.

### p(adj) < 0.001.

**Table 6:** Pairwise comparisons of endurance running performance (1,000 m for males, 800 m for females) by gender and grade (dunn's test with Bonferroni correction)

Gender	Grade	CF VS Control			CFS VS Control			CFS VS CF		
		*Z*	*P*	95%CI	*Z*	*P*	95%CI	*Z*	*P*	95%CI
Male	Grade7	−2.353	0.056	(37, 61)	−1.424	0.463	(15, 77)	−0.506	1	(−87, 36)
	Grade8	5.876	<0.001***	(22, 77)	3.453	0.002**	(24, 68)	0.736	1	(−66, 49)
	Grade9	3.519	0.001**	(9, 45)	3.571	0.001**	(21, 61)	1.559	0.357	(−39, 54)
	Grade10	7.888	<0.001***	(30, 48)	4.991	<0.001***	(30, 63)	1.133	0.772	(−9, 30)
	Grade11	6.614	<0.001***	(20, 38)	4.381	<0.001***	(23, 53)	0.822	1	(−16, 31)
	Grade12	2.796	0.016*	(4, 29)	3.923	<0.001***	(20, 49)	2.33	0.059	(−14, 37)
	Cram School	—	—	—	—	—	—	—	—	—
Female	Grade7	−0.631	1	(43, 56)	0.242	1	(72, 108)	0.465	1	(14, 64)
	Grade8	8.128	<0.001***	(45, 63)	5.366	<0.001***	(85, 99)	0.49	1	(16, 53)
	Grade9	1.105	0.808	(10, 33)	−1.065	0.861	(31, 59)	−1.471	0.424	(−1, 44)
	Grade10	−0.333	1	(52, 68)	1.188	0.704	(56, 78)	1.224	0.663	(−12, 19)
	Grade11	−2.214	0.08*	(21, 29)	2.128	0.1	(66, 89)	2.937	0.01^#^	(43, 66)
	Grade12	0.94	1	(17, 27)	0.033	1	(35, 65)	−0.417	1	(13, 43)
	Cram School	−3.082	0.006**	(16, 33)	1.281	0.6	(22, 58)	2.851	0.013^#^	(−4, 35)

Pairwise comparisons were conducted using Dunn's test with Bonferroni adjustment, Bonferroni-adjusted *p* values are reported. *p*_adj < 0.05 was considered statistically significant. When compared with the control group; compared with the CF group.

* *p*(adj) < 0.05.

** *p*(adj) < 0.01.

*** *p*(adj) < 0.001; compared with the CF group.

# *p*(adj) < 0.05.

Tables 3 and 4 results indicate that in the comparison of vital capacity test scores, only the CF and CFS groups of female students from the cram school demonstrated significantly lower vital capacity test scores than the control group (*p* < 0.05). No significant differences were observed in comparisons between the remaining groups. Furthermore, Table 5 shows that in endurance running comparisons, the 1,000 m run test results for both CF and CFS groups of male students from grade 10 to grade 12 were significantly lower than those of the control group to varying degrees (*p* < 0.01 & 0.001). while the 800 m run test results for both the CF and CFS groups of female students across all grades, including those from the cram school, were significantly lower than those of the control group (*p* < 0.01 & 0.001). These findings indicate that CFS levels were associated with poorer endurance running performance to varying degrees, particularly among female students. Further post hoc analysis (Table 6) indicated that senior high school grade 12 male students in the CFS group achieved significantly lower 1,000 m run test scores than their counterparts in the CF group (*p* < 0.05). Similarly, female students in the CFS group across both senior high school grade 11 and grade 12 achieved significantly lower 800 m run test scores than those in the CF group (*p* < 0.05 & 0.001). This results suggest that poorer endurance running performance was associated with higher CFS severity in these grade groups.

## 4 Discussion

Chronic fatigue syndrome (CFS) in adolescents has been associated with reduced quality of life (17, 18). Early intervention for CFS in this age group is crucial, yet there is currently no universally accepted standard for screening adolescents. While researchers commonly utilize the U.S. Centers for Disease Control and Prevention (CDC-1994) criteria developed for adults, this study focuses on secondary school students. Given the unique characteristics of adolescents, we have adapted the screening criteria proposed by Shi Jieyao's team at Soochow University for Chinese adolescents (6). Secondary school pupils, undergoing significant physiological and psychological changes, are exposed to various external pressures, including academic demands, parental expectations, and peer competition (19). Research indicates that prolonged chronic stress has been associated with CFS-like symptoms (20). Through a sample survey of 25 secondary schools in Shaanxi Province, this study found an overall CFS detection rate of 2.059% among students, significantly higher than the average levels reported in previous studies across different populations and diagnostic criteria (21).

Research consistently shows distinct characteristics in the distribution of CFS across various population groups, with a notably higher positive detection rate in women compared to men, usually at a ratio ranging from 2:1 to 4:1 (22), The detection rate of CFS was descriptively higher in female students (2.234%) than in male students (1.903%), although this difference did not reach statistical significance (*χ*^2^ = 1.210, *p* = 0.546). This gender disparity may stem from women's greater tendency to internalise stress and report more physical and emotional symptoms (23). Notably, CFS detection rate exhibited an increasing trend from grade 7 to cram schools, with students in cram schools showing a detection rate as high as 3.728%. As educational institutions specifically admitting candidates who failed the national university entrance examination, cram schools place students not only under the pressure of retaking the examination but also confronting the frustration of prior failure. This academic context may be associated with greater psychological burden (24), which may partly explain the elevated CFS screening detection rate observed among students in cram schools.

This study observed a significant association between CFS severity and endurance-related physical fitness performance in Secondary school students. Comparative analysis revealed a weak to moderate negative correlation between CFS and the 1,000-meter run times for boys and the 800-meter run times for girls across all grade levels. Poor performance in endurance tests may objectively indicate physical fatigue in students. Aerobic endurance in adolescents is considered a key indicator of their cardiopulmonary function and overall health status (25), while the physiological characteristics of CFS include immune system dysfunction, chronic inflammation, autonomic nervous system dysfunction, and abnormalities in cellular energy metabolism (26, 27). These changes are interrelated with students' physical health levels and their athletic abilities, particularly aerobic exercise capacity (28). From another perspective, physical fitness levels, particularly aerobic endurance, reflect an individual's reserve capacity to cope with physiological and psychological stressors (29). Studies indicate that students with poorer physical fitness may have lower physiological reserve, and previous studies have suggested that such conditions may be associated with greater vulnerability to fatigue-related symptoms under academic or emotional stress. However, the present cross-sectional study cannot determine the direction of this association (30), which aligns with the findings of this study. Regular physical exercise has been shown to regulate immune function, improve mood, and enhance stress resistance (31). Therefore, regular physical exercise and better physical fitness may be associated with greater physiological resilience and lower levels of CFS-like symptoms, although longitudinal studies are needed to confirm this relationship.

In summary, CFS is a widespread health issue among secondary school students, closely associated with high academic pressure and low physical endurance. The findings of this study not only provide evidence regarding the screening detection rate and associated factors of CFS-like symptoms in this population, but also offer important guidance for the development of targeted public health policies and clinical intervention strategies.

### 4.1 Strengths and limitations

The study's strengths include its substantial sample size, focus on Chinese secondary school students—a group with limited research attention, and a study design integrating self-reported CFS symptoms with objective physical health measures. However, limitations exist. The cross-sectional design precludes establishing causality between CFS-like symptoms and declining physical health, and using the CDC-1994 standard questionnaire for CFS assessment represents a screening-based classification, not a clinical diagnosis, potentially introducing bias. Moreover, Significant methodological limitations exist, notably due to the lack of adjustment for clustering effects when drawing samples from various schools, potentially impacting variance estimation and the reliability of statistical inferences.

In conclusion, although the research was stratified by gender and grade, it failed to adequately consider the characteristics at the school level. Unmeasured confounding factors such as sleep quality, psychological stress, and nutritional status may also affect the relationships between the variables. Future research should include data collection at the school level and multilevel modeling. It is recommended to conduct longitudinal cohort studies and adopt a two-stage approach: first, conduct a large-scale screening, and then conduct systematic clinical assessment and diagnosis of positive cases to determine the detection rate of chronic fatigue syndrome (CFS) among adolescents. At the same time, it is recommended to integrate biological measurement data, such as immune indicators (32), metabolic profiling (33), to comprehensively explore the pathogenesis of chronic fatigue syndrome in adolescents and its relationship with physical health.

## 5 Conclusions

Chronic fatigue syndrome-like symptoms among secondary school students in Shaanxi Province warrant attention. In this cross-sectional study, the screening detection rate of CFS tended to increase with grade level and was highest among students in cram schools. Higher CFS levels were associated with poorer physical fitness achievement, particularly in endurance running events, including the 1,000-m run in boys and the 800-m run in girls. These findings suggest that students with CFS-like symptoms may require additional attention in school health monitoring, especially with respect to endurance-related fitness. However, because of the cross-sectional design, the observed associations should not be interpreted as evidence of causality. Future longitudinal studies incorporating clinical confirmation of CFS, school-level clustering adjustment, and potential confounders such as sleep quality, socioeconomic status, psychological stress, and physical activity are needed to clarify the direction and mechanisms of these associations.

## Data Availability

The original contributions presented in the study are included in the article/Supplementary Material, further inquiries can be directed to the corresponding author/s.

## References

[1] Prins JB van der Meer JW Bleijenberg G. Chronic fatigue syndrome. Lancet (London, England). (2006) 367(9507):346–55. 10.1016/S0140-6736(06)68073-2 16443043

[2] Bowlus CL Arrivé L Bergquist A Deneau M Forman L Ilyas SI. AASLD practice guidance on primary sclerosing cholangitis and cholangiocarcinoma. Hepatology. (2022) 77(2):659–702. 10.1002/hep.32771 36083140

[3] Prajjwal P Kalluru PKR Marsool MD Inban P Gadam S Al-ezzi SMS. Association of multiple sclerosis with chronic fatigue syndrome, restless legs syndrome, and various sleep disorders, along with the recent updates. Ann Med Surg. (2023) 85(6):2821–32. 10.1097/MS9.0000000000000929 PMC10289738 37363482

[4] Tański W Wójciga J Jankowska-Polańska B. Association between malnutrition and quality of life in elderly patients with rheumatoid arthritis. Nutrients. (2021) 13(4):1259. 10.3390/nu13041259 33921207 PMC8070444

[5] Yeager DS Bryan CJ Gross JJ Murray JS Krettek Cobb D H. F. Santos P. A synergistic mindsets intervention protects adolescents from stress. Nature. (2022) 607(7919):512–20. 10.1038/s41586-022-04907-7 35794485 PMC9258473

[6] Shi JY Xu Y. Status of chronic fatigue and the influencing factors among middle school students. Chin J Sch Health. (2014) 35(7):1017–9.

[7] Xia Q Han X Zheng WW Dai Y. Analysis of the current situation and influencing factors of chronic fatigue syndrome among middle school students in Yangpu district, Shanghai. Chin J Sch Health. (2014) 35(6):918–20.

[8] The General Office of the Central Committee of the Communist Party of China and the General Office of the State. Opinions on further reducing the homework burden and extracurricular training burden of students in the compulsory education stage (2021).

[9] Shu ZG. Construction of Risk Assessment Index System for Middle School Students’ Sports Activities (MA thesis). Chengdu Sports University, Chengdu (2022).

[10] Stierwalt H McCalley A McCoin C Thyfault JP. Interactions between statins, exercise, and health: a clinical update. J Clin Exerc Physiol. (2022) 11(2):54–61. 10.31189/2165-6193-11.2.54

[11] Spinicci M Graziani L Tilli M Nkurunziza J Vellere I Borchi B. Infection with SARS-CoV-2 variants is associated with different long COVID. Phenotypes. Viruses. (2022) 14(11):2367. 10.3390/v14112367 36366465 PMC9698829

[12] Chen P. Physical activity, physical fitness, and body mass index in the Chinese child and adolescent populations: an update from the 2016 physical activity and fitness in China-the youth study. J Sport Health Sci. (2017) 6(4):381–3. 10.1016/j.jshs.2017.09.011 30356661 PMC6189246

[13] Keller B Receno CN Franconi CJ Harenberg S Stevens J Mao X. Cardiopulmonary and metabolic responses during a 2-day CPET in myalgicencephalomyelitis/chronic fatigue syndrome: translating reduced oxygen consumption to impairment status to treatment considerations. J Transl Med. (2024) 22:627. 10.1186/s12967-024-05410-5 38965566 PMC11229500

[14] Denadai B Greco C. Could middle- and long-distance running performance of well-trained athletes be best predicted by the same aerobic parameters? Curr Res Physiol. (2022) 5:265–9. 10.1016/j.crphys.2022.06.006 35800136 PMC9253837

[15] Fukuda K Straus SE Hickie I Sharpe MC Dobbins JG Komaroff A. The chronic fatigue syndrome: a comprehensive approach to its definition and study. Ann Intern Med. (1994) 121(12):953–9. 10.7326/0003-4819-121-12-199412150-00009 7978722

[16] Ministry of Education. Notice of the Ministry of Education on Issuing the “National Student Physical Health Standard (Revised in 2014)”. Beijing: Ministry of Education of the People’s Republic of China Government Portal (2014).

[17] Dantoft TM Ebstrup JF Linneberg A Skovbjerg S Madsen AL Mehlsen J. Cohort description: the Danish study of functional disorders. Clin Epidemiol. (2017) 9:127–39. 10.2147/CLEP.S129335 28275316 PMC5333638

[18] Nater UM Heim CM Raison C. Chronic fatigue syndrome. Handb Clin Neurol. (2012) 106:573–87. 10.1016/B978-0-444-52002-9.00034-6 22608645

[19] Aziz M Chemnad K Al-Harahsheh S Abdelmoneium AO Baghdady A Ali R. Depression, stress, and anxiety versus internet addiction in early and middle adolescent groups: the mediating roles of family and school environments. BMC Psychol. (2024) 12:184. 10.1186/s40359-024-01659-z 38570890 PMC10993579

[20] Liu T Sun W Guo S Chen T Zhu M Yuan Z. Research progress on pathogenesis of chronic fatigue syndrome and treatment of traditional Chinese and western medicine. Auton Neurosci. (2024) 255:103198. 10.1016/j.autneu.2024.103198 39047501

[21] Lim E-J Ahn Y-C Jang E-S Lee S-W Lee S-H Son C-G. Systematic review and meta-analysis of the prevalence of chronic fatigue syndrome/myalgic encephalomyelitis (CFS/ME). J Transl Med. (2020) 18:100. 10.1186/s12967-020-02269-0 32093722 PMC7038594

[22] Kapur N Webb R. Suicide risk in people with chronic fatigue syndrome. Lancet (London, England). (2016) 387(10028):1596–7. 10.1016/S0140-6736(16)00270-1 26873809

[23] Pinquart M Shen Y. Depressive symptoms in children and adolescents with chronic physical illness: an updated meta-analysis. J Pediatr Psychol. (2010) 36(4):375–84. 10.1093/jpepsy/jsq104 21088072

[24] Babacan T. Evaluation of impact of the high school entrance exam on students and parents according to meaningful evaluation model. Cukurova Univ Fac Educ J. (2024) 53:718–54. 10.14812/cuefd.1357064

[25] Kesäniemi Y Danforth E Jensen M Lefèbvre P Reeder BA. Dose-response issues concerning physical activity and health: an evidence-based symposium. Med Sci Sports Exerc. (2001) 33(6):S351–8. 10.1097/00005768-200106001-00003 11427759

[26] Walitt B Singh K LaMunion SR Hallett M Jacobson S Chen K. Deep phenotyping of post-infectious myalgic encephalomyelitis/chronic fatigue syndrome. Nat Commun. (2024) 15:907. 10.1038/s41467-024-45107-3 38383456 PMC10881493

[27] Demitrack M Dale J Straus S Laue L Listwak SJ Kruesi MJP. Evidence for impaired activation of the hypothalamic-pituitary-adrenal axis in patients with chronic fatigue syndrome. J Clin Endocrinol Metab. (1991) 73(6):1224–34. 10.1210/jcem-73-6-1224 1659582

[28] Li T Xu Q Wang S Qi K Su P Silva RM. Effects of recreational small-sided games from different team sports on the improvement of aerobic fitness in youth sedentary populations: a systematic review. Heliyon. (2023) 9(11):e22041. 10.1016/j.heliyon.2023.e22041 38045141 PMC10689881

[29] Mas-Bargues C Viña J Borrás C. Editorial: frailty- and age-associated diseases: possibilities for intervention. Front. Aging. (2024) 5:1522451. 10.3389/fragi.2024.1522451 39669433 PMC11634792

[30] Hickie I Davenport T Wakefield D Vollmer-Conna U Cameron B Vernon SD. Post-infective and chronic fatigue syndromes precipitated by viral and non-viral pathogens: prospective cohort study. Br Med J. (2006) 333:575. 10.1136/bmj.38933.585764.AE 16950834 PMC1569956

[31] Pescatello LS Franklin BA Fagard R Farquhar WB Kelley GA Ray CA. American College of Sports Medicine position stand. Exercise and hypertension. Med Sci Sports Exerc. (2004) 36(3):533–53. 10.1249/01.mss.0000115224.88514.3a 15076798

[32] Klimas NG Salvato FR Morgan R Fletcher MA. Immunologic abnormalities in chronic fatigue syndrome. J Clin Microbiol. (1990) 28:1403–10. 10.1128/jcm.28.6.1403-1410.1990 2166084 PMC267940

[33] Naviaux RK Naviaux JC Li K Bright AT Alaynick WA Wang L. Metabolic features of chronic fatigue syndrome. Proc Natl Acad Sci USA. (2016) 113(37):E5472–80. 27573827 10.1073/pnas.1607571113 PMC5027464

